# Effects on perceived pain and somatosensory function after transcutaneous neuromodulation in patients with chronic low back pain: a quasi-experimental study with a crossover intervention

**DOI:** 10.3389/fpain.2025.1525964

**Published:** 2025-04-15

**Authors:** Francisco Selva-Sarzo, Eleuterio A. Sánchez Romero, Juan Nicolás Cuenca-Zaldívar, Beatriz García-Haba, Claudio Akiyama, Rob Sillevis, Samuel Fernández-Carnero

**Affiliations:** ^1^ Francisco Selva Physiotherapy Clinic, Valencia, Spain; ^2^ Physiotherapy Faculty, Universitat de València, Valencia, Spain; ^3^ Interdisciplinary Research Group on Musculoskeletal Disorders, Madrid, Spain; ^4^ Department of Rehabilitation Sciences, Florida Gulf Coast University, Fort Myers, FL, United States; ^5^ Physiotherapy and Orofacial Pain Working Group, Sociedad Española de Disfunción CraneomandibularyDolor Orofacial (SEDCYDO), Madrid, Spain; ^6^ Research Group in Nursing and Health Care, Puerta de Hierro Health Research Institute-Segovia deArana (IDIPHISA), Majadahonda, Spain; ^7^ Facultad de Enfermería y Fisioterapia, Departamento de Fisioterapia, Grupo de Investigación en Fisioterapia y Dolor, Universidad de Alcalá, Alcalá de Henares, Spain; ^8^ Physical Therapy Unit, Primary Health Care Center “El Abajón”, Las Rozas de Madrid, Spain; ^9^ Facultad de Ciencias de la Salud, Carrera en Licenciatura en Kinesiología y Fisiatría, Cátedra de Fisioterapia, Universidad de Flores, CABA, Argentina; ^10^ Fundación Barceló, Facultad de Medicina, Carrera en Licenciatura en Kinesiología y Fisiatría, Cátedra de Fisiología Humana, Instituto Universitario de Ciencias de la Salud, Santo Tomé, Corrientes, Argentina; ^11^ School of Kinesiology, Universidad de Flores, Asociación Argentina Para el Estudio del Dolor (IASP Official Argentina Chapter), Buenos Aires, Argentina

**Keywords:** somatosensory system, low back pain, magnetic fields, epidermis, neuromodulation therapy

## Abstract

**Background:**

Transcutaneous magnetic neuromodulation is a noninvasive technique that may influence pain perception and mobility by modulating epidermal afferents and autonomic nervous system activity. However, its effects on chronic non-specific low back pain (CNSLBP) remain unclear.

**Objective:**

This study evaluated the effects of transcutaneous neuromodulation applied to the lumbar spine on the pressure pain threshold (PPT) and ankle dorsiflexion range of motion (DROM) in patients with chronic non-specific low back pain.

**Methods:**

A single-group prospective cohort study with crossover intervention was conducted from June to December 2021. A convenience sample of 39 patients with CNSLBP was included in this study. Each participant received two interventions in a randomized sequence: transcutaneous neuromodulation tape with magnetic particles (TMP) and placebo kinesiology tape (KT). A one-week washout period was implemented between the interventions. TMP was applied at the lumbar spinal levels for 48 h, following standard recommendations for neuromodulation frequency (constant exposure via magnetic particles), intensity (low-energy field), and time (continuous exposure over two days). The primary outcome measure was PPT assessed using algometry, and the secondary outcome was DROM assessed using the Lunge Test. This study adhered to the STROBE guidelines for observational studies.

**Results:**

The Lunge test revealed no significant group–time interaction [F(2, 152) = 0.132, *p* = 0.752], with a small effect size [F(1, 76) = 0.699, *p* = 0.406]. The main effect group showed a small non-significant effect [$\eta_{p}^{2}$ = 0.009 (0, 0.091)]. However, the main effect of time was significant [F(2, 152) = 147.669, *p* = 0.001] with a large effect size [$\eta_{p}^{2}$  = 0.66 (0.54, 0.735)]. Pairwise leg comparisons were not significant (*p* > 0.05). For the pressure pain threshold, significant differences (*p* < 0.05) with moderate to large effect sizes were observed. PPTs varied by vertebral level, with significant differences in site-specific comparisons between specific levels.

**Conclusions:**

Transcutaneous neuromodulation using TMP applied to the lumbar spine reduces perceived pain and increases ankle dorsiflexion range of motion in patients with CNSLBP. These findings suggest that epidermal afferent modulation may contribute to pain relief and motor function enhancement, providing a novel approach for noninvasive pain management.

## 1 Introduction

Despite significant advances in the understanding of musculoskeletal pain and the wide variety of available therapeutic approaches, the global burden of persistent pain continues to rise, placing immense pressure on healthcare systems worldwide (1). The neurophysiological mechanisms of pain processing involve three primary centers: the pregenual anterior cingulate cortex (pgACC), dorsal anterior cingulate cortex (dACC), and somatosensory cortex (SSC), corresponding to the descending, medial, and lateral pain pathways (2). The descending pain inhibitory system, which is crucial for context-dependent pain modulation and placebo analgesia, is often impaired in widespread pain syndromes such as fibromyalgia (3). Pain emerges from an imbalance between ascending nociceptive input and descending inhibitory control, leading to dysregulated autonomic nervous system (ANS) responses (4). There is evidence that epidermal stimulation is correlated with ANS function, making it a relevant factor in chronic pain assessment, particularly through pupillometry (2, 5).

Quantitative Sensory Testing (QST) is widely used to assess somatosensory function by measuring the responses to mechanically controlled stimuli (6, 7). The pressure algometry test is one of the most commonly used methods for evaluating pain sensitivity (8, 9). Structural changes in intraepidermal nerve fibers have been proposed as biomarkers of maladaptive brain plasticity following peripheral nerve degeneration, contributing to neuropathic pain (10). The epidermis, derived embryologically from the ectoderm like the central nervous system, plays a crucial role in pain perception through cutaneous sensory and autonomic nerve fibers (11, 12). Merkel cells and keratinocytes respond to mechanical stimuli, forming a tactile dome with Aβ nerve endings that modulate sensory transduction (13).

The epidermis also serves as a key interface for pain modulation via neuroimmune and endocrine signaling (5, 13–21). Ultraviolet B (UVB) radiation directly affects epidermal cells, such as Langerhans cells, keratinocytes, and melanocytes, thereby influencing nociceptive pathways through A*δ* and C fibers (13–21). Despite their distinct functions, these epidermal components operate within a complex network that regulates sensory inputs, inflammation, and neuroendocrine interactions (14–21).

Given its integral role in pain perception, the epidermis has become a target for novel neuromodulation strategies including transcutaneous magnetic neuromodulation. Tape with magnetic particles (TMP), an adhesive tape embedded with microparticles that generate weak magnetic fields, has demonstrated both local and systemic effects on sensory afferents (5, 22, 23). It has been suggested that TMP may modulate Aδ and C fibers systemically, with secondary effects on Aβ fibers locally, potentially altering pain processing and muscle function (5, 22, 23).

Placebo-controlled studies indicate that kinesiology tape (KT) may offer some benefit in reducing pain and disability in chronic nonspecific low back pain (CNSLBP) (24). Moreover, reduced ankle dorsiflexion range of motion (DROM) has been identified as a risk factor for lower extremity injuries and is commonly assessed during rehabilitation using the Ankle Lunge Test (ALT) (25, 26). Given that CNSLBP affects over 540 million individuals worldwide and poses a substantial socioeconomic burden, alternative non-invasive approaches targeting the epidermal afferent nervous system warrant further investigation.

Neuromodulation techniques aim to influence the pain pathways through various physical stimuli. Unlike electrical neuromodulation, which directly stimulates nerves, transcutaneous magnetic neuromodulation affects neural and muscular functions by altering the electromagnetic properties. Recent findings have suggested that TMP may enhance neuromuscular responses and reduce pain sensitivity in musculoskeletal conditions (5, 22, 23). However, the mechanism underlying these effects remains unclear.

This study aimed to evaluate the effects of transcutaneous neuromodulation applied to the lumbar spine to reduce pain and increase ankle dorsiflexion range of motion in patients with chronic nonspecific low back pain (CNSLBP).

## 2 Methods

### 2.1 Study design

A single-group prospective cohort study with a crossover intervention with three measurement points (baseline, experimental tape, and placebo tape) was conducted from June to December 2021 to assess the effects of transcutaneous neuromodulation applied to the lumbar spine to reduce pain and increase ankle dorsiflexion range of motion in patients with chronic non-specific low back pain (CNSLBP). The study was conducted in accordance with the Declaration of Helsinki and approved by the Ethical Committee of the University of Valencia (number 1315365). Informed consent was obtained from all subjects involved in the study. The procedures were conducted according to the Strengthening the Reporting of Observational Studies in Epidemiology (STROBE) statement and checklist (27).

### 2.2 Participants

For this study, a convenience sample of patients with CNSLBP was selected from different private practices in the city of Valencia (Spain): Acuario Sports Clinic, Aston Clinic specializing in traumatology and sports physiotherapy, Vanesa Vallet Clinic, and Francisco Selva Physiotherapy Clinic.

**Figure 1:**
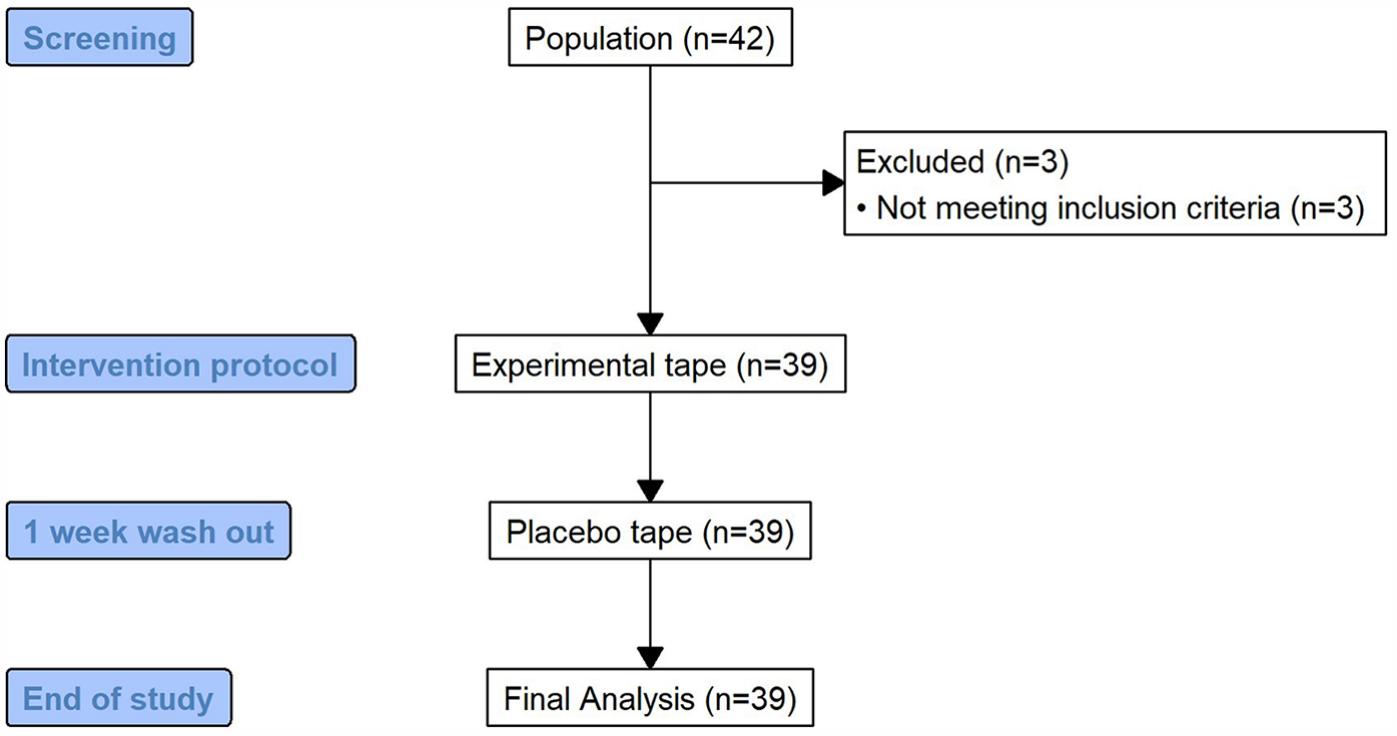
Study flow chart. Flow diagram depicting the recruitment process, exclusion criteria, and final sample size (*n* = 39) of patients included in the crossover study.

From an initial sample of 42 patients, 39 were finally included in the study (Figure 1); three participants were excluded: one due to a neurological condition affecting motor function and two due to contraindications to electromagnetic fields (implanted medical devices). Consequently, 39 participants were included and completed all phases of the study, with the following inclusion criteria: CNSLBP lasting more than three months, confirmed by a physician, aged between 18 and 65 years, residing in Valencia (Spain), diagnosed by a physician, and ability to understand and provide informed consent in Spanish. The exclusion criteria were as follows: allergy or contraindications to adhesive tape; pregnancy; contraindications to electromagnetic fields, including pacemakers or implanted medical devices; and neurological conditions that cause motor impairment or sensory deficits unrelated to CNSLBP, such as multiple sclerosis, spinal cord injury, peripheral neuropathy, or use of medications that may interact with magnetic fields. Participants were diagnosed with CNSLBP according to the clinical guidelines of the European Spine Society and the National Institute for Health and Care Excellence (NICE) (28). If the participants met the study criteria, they provided written consent to participate in the study. Demographic and preintervention data were also collected.

Two conditions were applied (experimental tape and placebo tape), with a washout period of one week between both.

### 2.3 Tests and measurements

Somatosensory function was assessed using Pressure Pain Threshold (PPT) testing at various spinal levels. PPT was measured using a Wagner Force Dial FDK 20 algometer by applying incremental pressure until the participant reported the first perception of pain. This method evaluates nociceptive processing and sensory modulation, offering insights into the changes in pain sensitivity following transcutaneous neuromodulation (with TMP).

The tape with magnetic particles (TMP) used was Magnetic Tape (Magnetic Tape®, S.L., Valencia, Spain), and the tape used as placebo was kinesiology tape (KT), an unbranded tape. Tests were performed at baseline and immediately after application of the experimental tape and one week of washout later, after application of placebo tape.

#### 2.3.1 Main outcome

**Figure 2:**
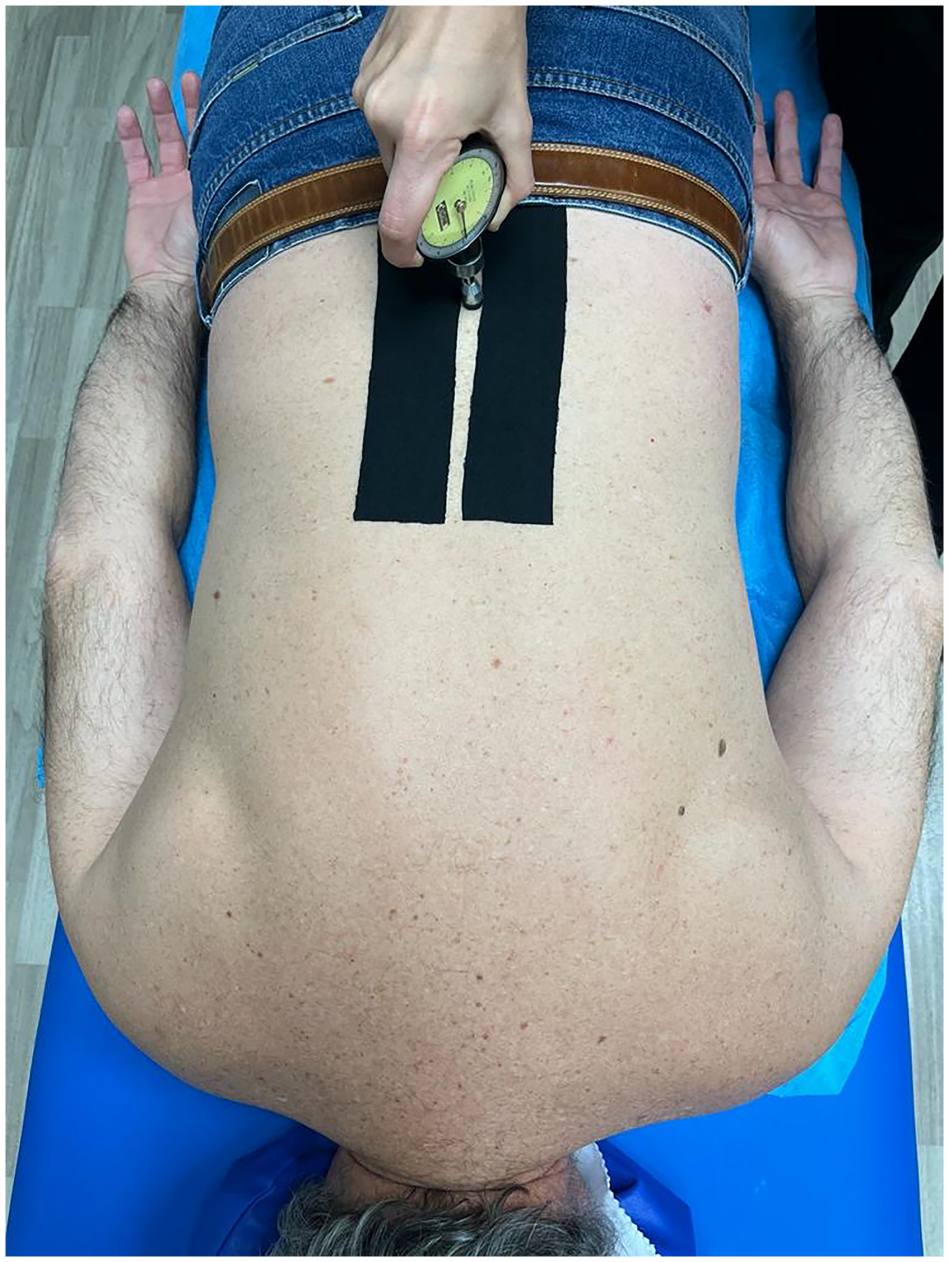
Pressure pain threshold measurement (PPT). Illustration of the algometry procedure using the Wagner Force Dial FDK 20 with a 1 cm^2^ probe over the lumbar spinous processes to assess maximum pressure pain threshold. Measurements were performed in prone position across lumbar levels.

The maximum pressure to achieve the pain threshold (PPT) was measured using a Wagner Force Dial FDK 20 algometer with a 1 cm^2^ footprint. PPT was assessed with the subjects in the prone position by applying posteroanterior pressure over the spinous processes (Figure 2) (5, 8, 9).

#### 2.3.2 Secondary outcomes

**Figure 3:**
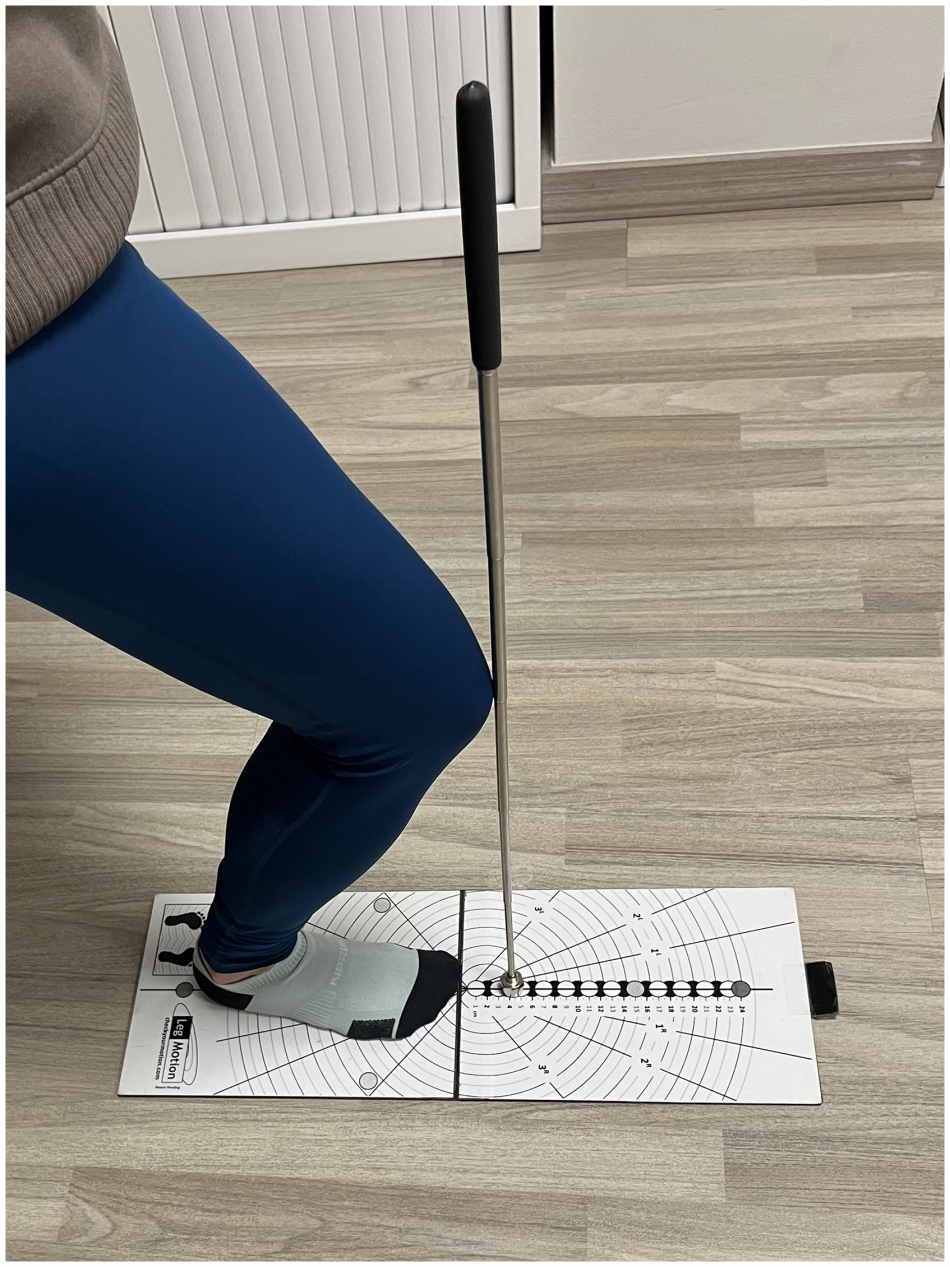
Lunge test measurement. Demonstration of the weight-bearing ankle dorsiflexion range of motion assessment using the LegMotion® platform. Measurements were taken for both ankles (*n* = 39) under each intervention condition.

PPT at 1 kg, 2 kg, and 3 kg were also measured. The Lunge Test was used to measure ankle mobility because of its reliability in measuring the ankle dorsiflexion range with weight bearing (26). The LegMOtion platform was used to measure the increase in dorsal flexion (Figure 3). The use of the LegMotion platform is validated and reliable for measuring the ankle dorsiflexion range of motion (29).

Before starting the investigation, a familiarization session was conducted so that the assessments were consistent in terms of pain intensity using the Numerical Pain Rating Scale (NPRS). To do this, an algometer was pressed on the posterior deltoid at 2, 4, and 6 kg of pressure and then at 1, 2, and 3 kg of pressure, and the NPRS ratings matched the pressure intensities. As previously documented in a published study, the same method was employed (5). Familiarization sessions will also be conducted to ensure that the lunge test is performed correctly and that the results have maximum inter-observer reliability.

The patient was placed in the prone position and the spinous process was pressed perpendicular to the vertebral body to assess possible irritation of the terminal branches of the medial and intermediate branches of the dorsal ramus at each level. Possible dysfunction of the medial branch as it passes through the tunnel formed by the mamilloaccessory ligament has also been assessed (30, 31). Using localized sensitivity techniques on the spinous processes (30, 31), the sensitivity or local pain of the posterior or dorsal branch of each level was assessed. Perceived pain with the PPT will be assessed on two different days, leaving one week of washout by pressing at each level of the spine with 1, 2, and 3 kg and the maximum tolerable pressure. During each press, the subject rated the perceived pain using the NPRS while in prone position. Three measurements of each pressure were performed at each level, and each measurement was separated by at least one minute to extract the mean.

Active joint movements of both ankles were assessed using the lunge test (32). The patient was placed in the standing position. Three dorsal flexion measurements of each ankle were obtained without lifting the heel off the ground and trying to flex the knee as far as possible, and the location of flexion was marked with a rod (Figure 3) (32).

### 2.4 Intervention

This study followed a single-group crossover design without randomization in which all participants underwent both TMP and KT interventions in a fixed sequence. TMP was applied first, followed by a one-week washout period after which KT was applied. Outcome measures (PPT and DROM) were assessed immediately after each intervention, allowing for intrasubject comparisons. A washout period was implemented to reduce potential carryover effects, ensuring that the response to each intervention could be evaluated independently. During the tests, the researcher and subjects were blinded because they could not recognize which tape was being tested, as both tapes had the same appearance as they were black. The tape was applied longitudinally over the paravertebral musculature as medially as possible to the spinous processes at the L1–L5 levels (32). There was 0% elongation of the tapes, so no tension was created when applying the tapes.

### 2.5 Blinding protocol and pressure levels justification

Both the TMP and KT had identical textures and colors, ensuring visual blinding for participants and assessors. A third researcher, who was not involved in the assessments, managed tape allocation to maintain double blinding. Participants were unaware of the specific properties of each tape and the assessors were blinded to the intervention sequence. This protocol minimizes detection and performance bias, thus enhancing the internal validity of the study.

The tape was applied to the lumbar area because the participants experienced pain during the metameric innervation. The bony relief of the spinous process at each level was located, pressure was applied at the same point, and the diameter of the PPT head on the spinous process was marked at each level. This mark ensured the reliability of the subsequent measurements.

### 2.6 Statistical analysis

Statistical analysis was performed using R Ver. 4.3.1. (R Foundation for Statistical Computing, Institute for Statistics and Mathematics, Welthandelsplatz 1, 1020 Vienna, Austria).

Due to the absence of similar previous studies, the sample size was calculated with the first 10 subjects recruited, using the average pain threshold at maximum pressure, with a Student's *t*-test for dependent samples between the application of the experimental tape and the control. The final power of the study was calculated by using the same procedure. The data from these 10 subjects were included in the final analysis because they were declared as an internal pilot study in the study design (33–35). The R package PWR was used (36, 37).

The level of significance was set at *P* < 0.05. The distribution of the quantitative variables was tested using the Shapiro–Wilk test. The quantitative variables are shown as median [interquartile range] and the qualitative variables as absolute and relative values (%).

In the case of the pressure pain threshold (PPT), the presence of significant differences between the pain reported on the 10-point numerical pain rating scale at maximum pressure and at 1 kg, 2 kg, and 3 kg throughout the measurements was evaluated, both with the average values of each block (cervical, thoracic, lumbar, and sacral) and at each vertebral level, using the Friedman test and defining the effect size with Kendall's W as small (<0.1), medium (0.1–0.3), and large (>0.3). Intra-group *post hoc* tests were performed using the Wilcoxon signed-rank test with Bonferroni correction.

In the Lunge test, the presence of significant differences between both legs throughout the measurements was evaluated using a non-parametric mixed ANOVA with two factors, between (groups) and within (measurements), with means truncated at 20%. The effect size was defined as partial eta squared ($\eta_{p}^{2}$) obtained by bootstrap and defined as small (<0.06), medium (0.06–0.14) and large (>0.14). For *post hoc* tests, the Mann–Whitney *U*-test was applied between groups or the Wilcoxon signed rank test within groups, depending on the significance of the omnibus tests, applying the Bonferroni correction in both cases.

#### 2.6.1 Justification for statistical approach and interpretation of SEM overlap

**Figure 4:**
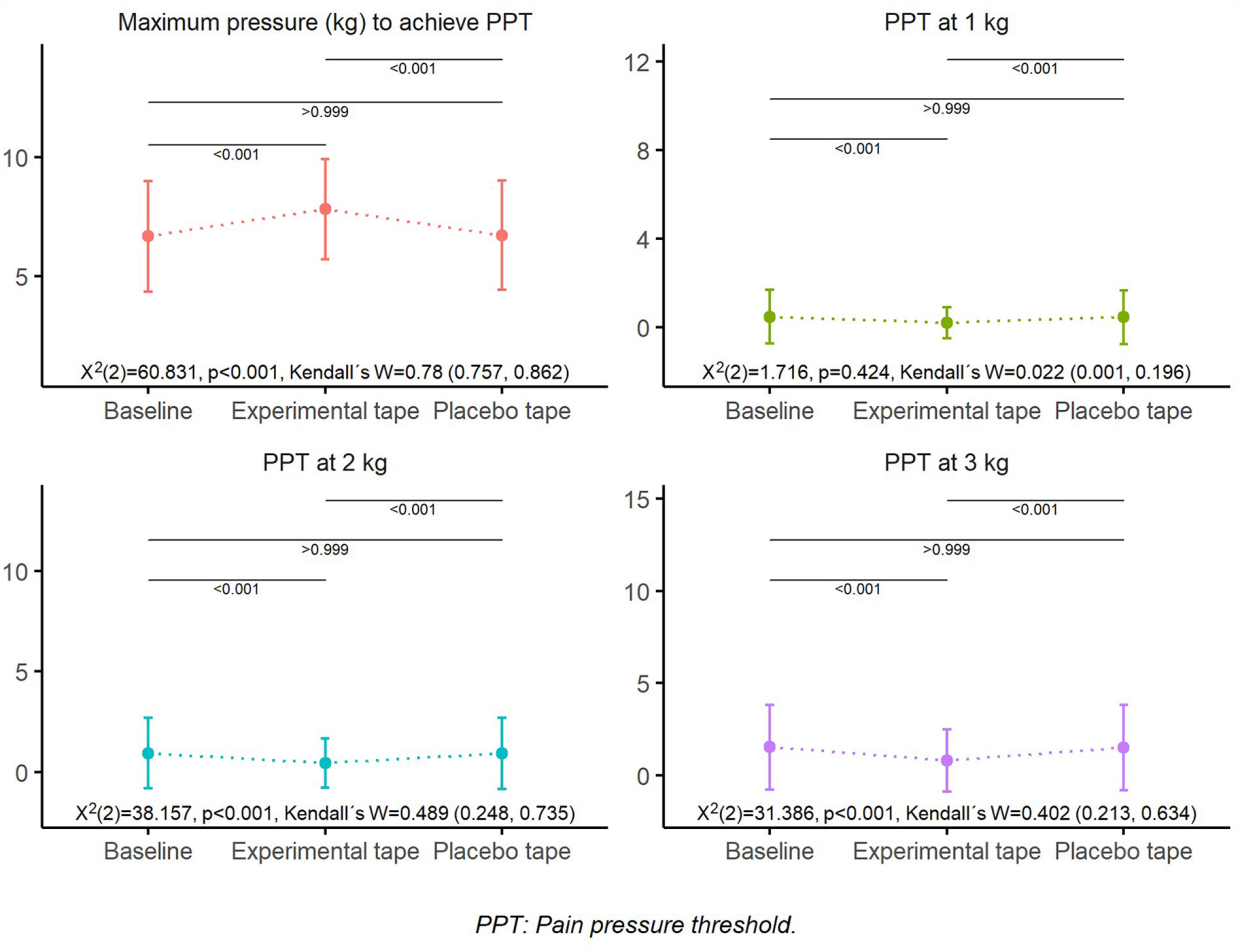
Overall pressure pain threshold (PPT). Mean PPT values at 1 kg, 2 kg, and 3 kg of applied pressure, and maximum pressure required to reach PPT across baseline, experimental tape, and placebo tape conditions (*n* = 39). Error bars represent standard error of the mean (SEM). The experimental tape significantly increased the maximum pressure required to reach PPT compared to baseline and placebo conditions.

Given the crossover design and within-subject repeated measures, non-parametric tests were used to evaluate intra-individual differences between baseline, placebo, and experimental conditions. Although Figure 4 presents error bars as standard error of the mean (SEM)—which may appear to overlap—statistical significance (*p* < 0.001) was detected using Friedman's test with Bonferroni-adjusted Wilcoxon signed-rank *post hoc* comparisons. These tests are specifically designed for intra-subject analysis and are sensitive to consistent directional changes, even when the absolute magnitude of difference is small. Furthermore, the high effect size values (e.g., Kendall's W = 0.78) support the robustness of the findings. The use of SEM in the graph reflects the precision of the mean, rather than the inter-subject variability, and may visually underestimate the significance of consistent trends observed within subjects.

### 2.7 Sample size

Accepting a risk α of 0.05, a power of 80% with an estimated effect size Cohen's D = 0.64 plus a 5% increase for non-normal distribution and a 10% increase for possible dropout, a total sample of 39 subjects was estimated.

## 3 Results

### 3.1 Participant enrollment and flow

A total of 42 patients were initially screened for eligibility. Of these, three patients were excluded because they did not meet the inclusion criteria (one due to a neurological condition affecting motor function and two due to contraindications to electromagnetic fields). Consequently, 39 participants were included in the study and completed all the phases of the intervention (Figure 1). No dropouts were recorded, and all the participants adhered to the study protocol.

### 3.2 Pain pressure threshold (PPT) outcomes

In the pressure pain threshold, significant differences (*p* < 0.05) were found in practically all variables, except in the global PPT with 1 kg (Figure 4), with significant effect sizes ranging from moderate to large (Table 1 and Supplementary Table S1). Significant differences (*p* < 0.05) were observed between the experimental and placebo tapes and between the experimental tape and baseline, with a higher maximum pressure to reach the PPT and lower PPT at 1, 2, and 3 kg in the experimental tape. In contrast, there were no significant differences (*p* > 0.05) between the placebo tape and baseline (Figure 4 and Supplementary Table S2).

**Table 1:** Pressure pain threshold averaged by vertebral region.

Measurement type	Baseline	Experimental tape	Placebo tape	*p*-value*	Kendall's W (95% CI)
Maximum pressure (kg) to achieve pressure pain threshold					
Cervical	4.77 [4.06, 6.49]	5.64 [4.99, 7.52]	4.88 [4.06, 6.45]	X2 (2) = 34.667, *p* < 0.001	0.444 (0.276, 0.686)
Thoracic	6.62 [4.74, 8.66]	8.00 [6.47, 9.53]	6.69 [4.80, 8.67]	X2 (2) = 38.263, *p* < 0.001	0.491 (0.309, 0.653)
Lumbar	6.75 [5.40, 8.14]	8.50 [6.97, 9.64]	6.78 [5.59, 8.36]	X2 (2) = 55.523, *p* < 0.001	0.712 (0.504, 0.856)
Sacrum	8.25 [6.34, 9.34]	9.46 [7.71, 10.00]	8.22 [6.34, 9.30]	X2 (2) = 50.908, *p* < 0.001	0.653 (0.467, 0.841)
Pressure pain threshold by kilogram					
Cervical 3 kg	1.20 [0.30, 2.50]	0.40 [0.00, 1.20]	1.20 [0.60, 2.30]	X2 (2) = 20.705, *p* < 0.001	0.265 (0.079, 0.519)
Cervical 2 kg	0.40 [0.10, 1.50]	0.00 [0.00, 0.50]	0.40 [0.20, 1.20]	X2 (2) = 22.16, *p* < 0.001	0.284 (0.073, 0.59)
Cervical 1 kg	0.00 [0.00, 0.80]	0.00 [0.00, 0.10]	0.00 [0.00, 0.70]	X2 (2) = 8.324, *p* = 0.016	0.107 (0.01, 0.269)
Thoracic 3 kg	0.75 [0.17, 1.83]	0.33 [0.00, 1.00]	0.75 [0.17, 1.88]	X2 (2) = 39.214, *p* < 0.001	0.503 (0.32, 0.704)
Thoracic 2 kg	0.33 [0.00, 1.08]	0.08 [0.00, 0.33]	0.42 [0.00, 1.00]	X2 (2) = 34.5, *p* < 0.001	0.442 (0.25, 0.657)
Thoracic 1 kg	0.00 [0.00, 0.80]	0.00 [0.00, 0.00]	0.08 [0.00, 0.38]	X2 (2) = 18.782, *p* < 0.001	0.241 (0.132, 0.36)
Lumbar 3 kg	1.80 [0.60, 2.50]	0.40 [0.00, 1.10]	1.80 [0.60, 2.40]	X2 (2) = 44.218, *p* < 0.001	0.567 (0.37, 0.794)
Lumbar 2 kg	0.80 [0.40, 1.50]	0.00 [0.00, 0.40]	1.00 [0.40, 1.40]	X2 (2) = 46.566, *p* < 0.001	0.597 (0.422, 0.755)
Lumbar 1 kg	0.00 [0.00, 0.80]	0.00 [0.00, 0.00]	0.20 [0.00, 0.60]	X2 (2) = 14.725, *p* = 0.001	0.189 (0.072, 0.363)
Sacrum 3 kg	0.75 [0.00, 1.50]	0.00 [0.00, 0.25]	0.75 [0.00, 1.50]	X2 (2) = 43.011, *p* < 0.001	0.551 (0.372, 0.76)
Sacrum 2 kg	0.00 [0.00, 0.50]	0.00 [0.00, 0.00]	0.25 [0.00, 0.62]	X2 (2) = 26.29, *p* < 0.001	0.337 (0.17, 0.48)
Sacrum 1 kg	0.00 [0.00, 0.00]	0.00 [0.00, 0.00]	0.00 [0.00, 0.00]	X2 (2) = 9.152, *p* = 0.01	0.117 (0.032, 0.27)

Data expressed with median [interquartile range]. 95% CI, 95% confidence interval.

* Significant if *p* < 0.05 (shown in red).

**Figure 5:**
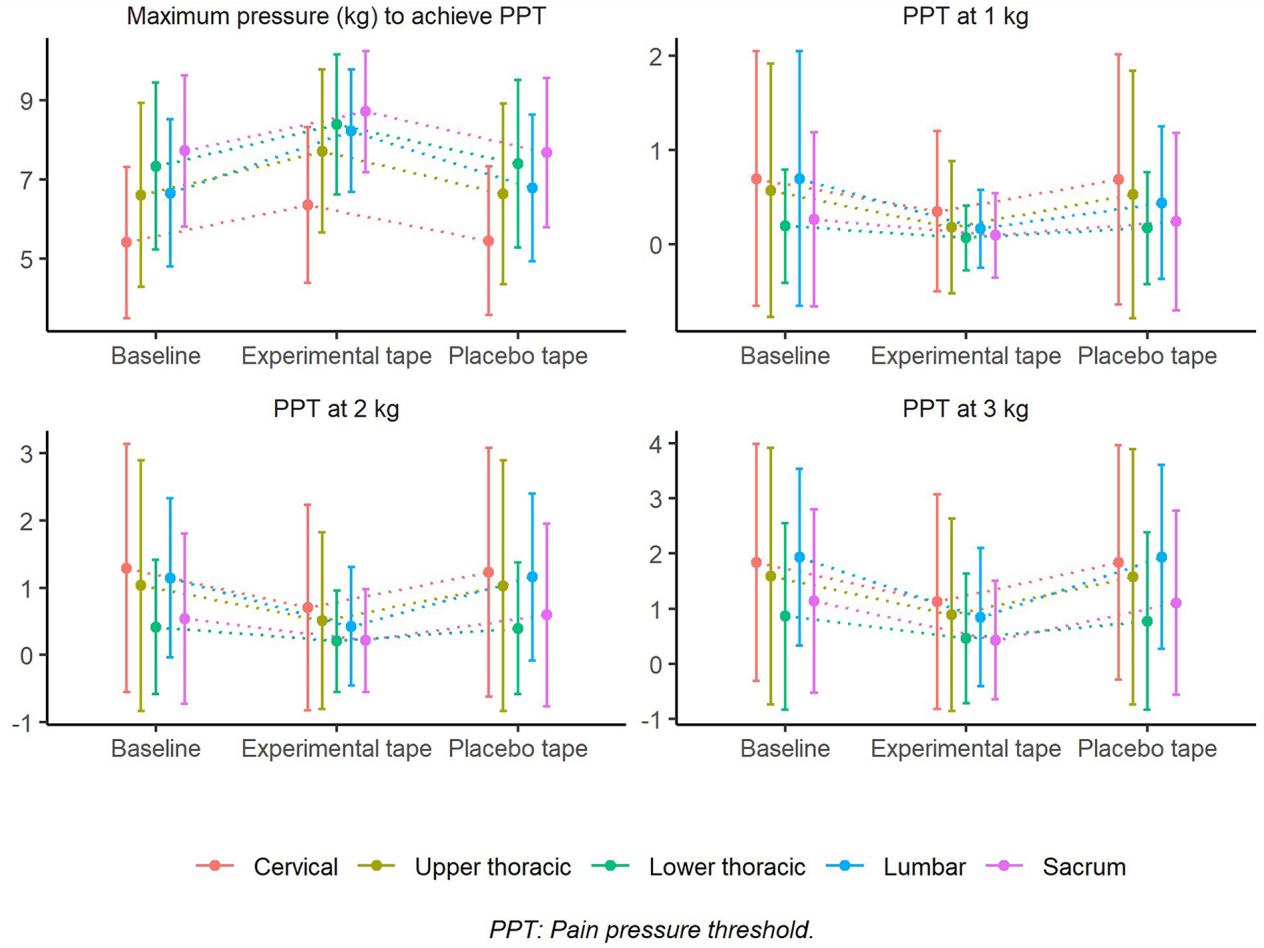
Pressure pain threshold (PPT) by vertebral region. PPT values at 1 kg, 2 kg, and 3 kg, and maximum pressure required to reach PPT, analyzed across vertebral regions (*n* = 39). Error bars represent standard deviation (SD). Statistical comparisons between conditions include *p*-values and Kendall's W effect sizes, showing significant differences between baseline, experimental tape, and placebo conditions.

The same pattern described above was maintained when the PPTs were compared by the vertebral region. At the same pressure (kg), the PPTs were systematically higher at the cervical and lumbar levels and lower at the sacral level; the PPTs were higher at the upper levels (T1–T8) compared at the lower (T9–T12). In contrast, the maximum pressure necessary to reach the PPT is greater at the lower vertebral level, being minimum at the cervical level and maximum at the sacral level, although it can be observed that at the lower thoracic level, it is greater than that at the lumbar level (Figure 5).

These differences were significant (*p* < 0.05), with no baseline differences between the lumbar vs. cervical levels on the experimental tape with 3 kg and the maximum pressure on the placebo tape, between the sacrum vs. cervical levels on the experimental tape with 1 kg and 3 kg, the maximum pressure on the placebo tape, between the sacrum and lower thoracic levels on the placebo tape with 1 kg, between the sacrum vs. lumbar levels on the experimental tape with 1 kg, between the upper thoracic vs. cervical levels on the experimental tape with 1 kg and 3 kg, between the upper thoracic vs. lower thoracic levels on the experimental tape with 3 kg, and between the upper thoracic vs. lumbar levels on the experimental tape with 1 kg (Supplementary Table S3).

### 3.3 Ankle dorsiflexion range of motion (Lunge test) outcomes

The Lunge test between both legs verified the absence of significant differences in the group:time interaction and main effect group (*p* > 0.05); however, there were significant differences in the main effect time with a large and significant effect size (Table 2). The *post hoc* tests in each leg showed significant differences (*p* < 0.05) in the right leg between all the measurement moments, with higher values in the experimental tape compared to the placebo tape and the baseline; in the left leg, there were significant differences between the experimental tape-baseline as between Placebo tape - Experimental tape with values always higher in the experimental tape, but not between the placebo tape - Baseline, among which the changes were not significant (*p* > 0.05) (Supplementary Table S4).

**Table 2:** Lunge test outome.

Leg	Baseline	Experimental tape	Placebo tape	Group (*p*-value*)	Time (*p*-value*)	Group:time (*p*-value*)	Group: time $\eta_{p}^{2}$ (95% CI)
Right leg	11.00 [9.00, 12.75]	12.00 [11.00, 14.00]	11.00 [9.75, 12.75]	F(1, 47.413) = 0.681, *p* = 0.413	F(2, 38.859) = 88.761, *p* < 0.001	F(2, 38.859) = 0.376, *p* = 0.689	0.008 (0, 0.034)
Left leg	11.00 [10.00, 13.00]	13.00 [11.50, 14.75]	11.50 [10.00, 13.00]				

Data expressed with median [interquartile range]. 95% CI, 95% confidence interval.

* Significant if *p* < 0.05 (shown in red).

### 3.4 Study power analysis

The final power of the study was 97.491%, with the maximum pressure required to achieve the PPT being above the minimum 80% required.

### 3.5 Parametric analysis confirmation

To confirm the robustness of our findings using an alternative approach, a repeated-measures ANOVA was also conducted for all main outcome variables (PPT at maximum pressure, 1 kg, 2 kg, 3 kg). The parametric analysis revealed results consistent with those of the non-parametric tests. Specifically, statistically significant differences were found in the maximum PPT [F(2, 76) = 14.72, *p* < 0.001], PPT at 2 kg [F(2, 76) = 8.56, *p* = 0.001], and PPT at 3 kg [F(2, 76) = 10.39, *p* < 0.001], confirming the previously observed intra-subject differences. No significant differences were observed at 1 kg [F(2, 76) = 1.12, *p* = 0.331], aligning with the non-parametric results. These findings reinforce the consistency of our data and indicate that the observed effects are not dependent on the statistical approach used.

## 4 Discussion

Our study demonstrated that transcutaneous neuromodulation with tape containing magnetic particles (TMP) applied to the lumbar spine significantly reduces perceived pain and increases ankle dorsiflexion range of motion in patients with chronic non-specific low back pain (CNSLBP). The results of this study showed changes after the intervention with a significant difference in the lunge test and posteroanterior algometry before and after lumbar treatment with TMP. These findings suggest a potential role for magnetic-based neuromodulation in musculoskeletal pain management, opening new avenues for noninvasive pain modulation strategies.

The findings of this study suggest that transcutaneous neuromodulation using TMP may influence pain perception and motor function through its interaction with the epidermal afferents and autonomic pathways. While this study did not conduct direct pathological examinations of local tissues, previous research has demonstrated that the epidermis plays a key role in sensory processing and neuro-immune interactions (13–21). The observed changes in pressure pain threshold (PPT) and dorsiflexion range of motion (DROM) following TMP application suggest that neuromodulation may extend beyond local mechanical effects, potentially altering nociceptive transmission and central pain modulation (4–6). Further studies incorporating neurophysiological and imaging techniques (e.g., fMRI and EEG) are warranted to validate these findings and elucidate the precise mechanisms underlying TMP's effects of TMP on pain modulation and sensorimotor function.

### 4.1 Interpretation of results in the context of current literature

In the epidermis, autonomic nerve fibers innervate melanocytes, Langerhans cells, and keratinocytes, which are influenced by electromagnetic fields, such as UV rays (13, 21). These structures form a part of the neuroimmunocutaneous system, which modulates pain and inflammation (13). Sensory information is transmitted via Aδ and C fibers, which may be influenced by TMP stimulation (38).

The epidermis is also crucial for the release of cytokines, chemokines, and neurohormones, allowing bidirectional communication with the nervous system (39). Given the common embryonic origins of the epidermis and nervous system, epidermal afferents may play a role in pain modulation (13, 39). Cutaneous free nerve endings are responsible for pain detection in the dorsal root ganglia (DRG), the primary site of nociceptive transmission (5, 40). Experimental models have shown that the inhibition of DRG neurons can significantly reduce nociception, suggesting a potential target for TMP modulation (5, 40).

Neuroimaging studies have further linked cutaneous nerve degeneration with altered thalamocortical connectivity in neuropathic pain, affecting areas such as the frontal cortex, cingulate gyrus, motor cortex, and the limbic system (10). These findings support the hypothesis that TMP may exert its effects through the modulation of central pain networks, rather than solely via local biomechanical influences.

Our findings contrast with those of studies evaluating kinesiology tape (KT), which have shown low-quality evidence for pain relief in chronic low back pain (24). This suggests that the neuromodulatory effects of TMP may be due to mechanisms beyond simple mechanical stimulation, likely involving neuroimmune interactions at the epidermal level (13, 21, 40).

### 4.2 Mechanisms underlying the effects of transcutaneous neuromodulation

Modulation of the autonomic nervous system (ANS) at the epidermal level has been proposed as a mechanism for pain regulation (2). Epidermal cells, which use the lateral spinothalamic tract, may be critical targets for neuromodulation (38, 41). TMP has been shown to positively influence ANS, which may explain its observed effects on pain reduction (5).

Ridder et al. (2) suggested that integrating systems neuroscience, autonomic nervous system science, network science, and neuroimmunology is crucial for understanding how acute pain transitions into chronic pain. Given that TMP modulates the perigenual anterior cingulate cortex (pgACC), a central hub of the descending pain inhibitory pathway, its effects may help restore the balance between the pain-facilitating and pain-inhibiting systems (42, 43).

Chronic pain conditions, including neuropathic pain, often involve maladaptive changes in descending pain control pathways (43). Our findings support this model, as TMP reduces perceived pain at painful levels while increasing the PPT across different spinal regions. These changes suggest that TMP may modulate pain perception by influencing the supraspinal networks.

### 4.3 Relationship between pain and movement dysfunction

Despite substantial advances in musculoskeletal pain research, the prevalence of chronic pain has not decreased (44). Our study suggests that epidermal dysfunction plays a crucial role in chronic pain.

Altered muscle activation patterns are common in chronic low back and affect locomotion and motor control (45). Tone disorders, often linked to dysfunction in descending motor pathways, can impair movement and lead to persistent pain (45–48). Central reflex mechanisms, rather than localized musculoskeletal dysfunction, may explain the limited mobility and persistent pain in some patients (45, 48).

The hip and ankle musculature are coactivated during movement (49–55), and patients with low back pain often show altered activation patterns between the tibialis anterior and transverse abdominis muscles (47). Reduced ankle dorsiflexion may be linked to dysfunction of the central descending reflexes or altered myotatic reflexes (54, 55). Our study found that TMP application led to immediate improvements in ankle dorsiflexion, suggesting an influence on central motor control mechanisms, rather than localized biomechanical effects.

Given these findings, TMP may act by inhibiting aberrant descending reflexes, thereby improving both pain perception and motor function. Future studies should explore whether TMP can be used as a neuromodulatory tool for movement rehabilitation in addition to pain relief.

### 4.4 Clinical implications and future directions

Our results suggest that TMP could be a valuable noninvasive intervention for CNSLBP, particularly in patients who continue to experience persistent pain despite conventional treatments. The observed modulation of both pain perception and motor function supports the need for further investigation of the potential mechanisms and broader applications of TMP in clinical settings.

One important area for future research is the evaluation of the long-term effects of TMP. Although this study demonstrated immediate pain relief and improvements in mobility, it remains uncertain whether these benefits are sustained over time or require repeated applications to maintain efficacy (5, 23). Understanding the duration and optimal frequency of TMP application could help refine the treatment protocols for chronic pain management.

In addition, comparisons between TMP and other neuromodulation techniques should be explored. Techniques such as electrical neuromodulation and vibration-based stimulation have been studied for their effects on pain modulation and sensorimotor function, and direct comparisons with TMP could provide insights into its relative effectiveness and unique neuromodulatory properties (24, 32). Investigating whether TMP offers distinct advantages or complementary effects compared with these existing modalities could help determine its location in multimodal pain management strategies.

Another critical avenue for future research involves neuroimaging studies, such as functional magnetic resonance imaging (fMRI), to examine how TMP influences pain-processing brain networks (10, 56–58). Understanding the cortical and subcortical mechanisms underlying TMP-induced pain relief could provide objective neurophysiological evidence for its effects and contribute to a more comprehensive model of pain modulation via epidermal afferents.

Further studies addressing these aspects could not only strengthen the clinical evidence supporting TMP but also help optimize its therapeutic application in patients with chronic pain conditions.

### 4.5 Limitations

Although this study provides valuable insights into the effects of transcutaneous neuromodulation with TMP on CNSLBP, several limitations must be acknowledged.

One of the main limitations is the absence of neurophysiological measures such as electroencephalography (EEG) or functional magnetic resonance imaging (fMRI), which would allow for a more comprehensive evaluation of TMP's effects on central pain modulation (56–58). Incorporating these techniques in future research could provide objective evidence of changes in neural activity associated with TMP application and further clarify its mechanism of action at cortical and subcortical levels.

Additionally, the study was limited to a short-term follow-up, focusing only on immediate effects after TMP application. As a result, it remains unclear whether these effects persist over time or whether repeated applications are necessary to maintain pain relief and mobility improvements. Future studies should include a longer follow-up period to assess the therapeutic benefits of TMP's therapeutic benefits (23).

Another limitation is related to the generalizability of the findings. This study was conducted in a specific population of individuals with CNSLBP, and the results may not be directly applicable to patients with other pain conditions or different demographic characteristics (55). Expanding research on diverse populations will help to determine whether TMP can be effectively integrated into broader pain management strategies.

Despite these limitations, this study provides preliminary evidence to support TMP as a potential neuromodulatory intervention for both pain management and movement rehabilitation. These findings highlight the need for further investigation of its long-term efficacy, neural mechanisms, and applicability in various clinical settings.

## 5 Conclusions

Transcutaneous neuromodulation using tape with magnetic particles applied to the lumbar spine reduces perceived pain and increases the range of motion of ankle dorsiflexion in patients with chronic non-specific low back pain.

These findings suggest that the dysfunction of movement that affects chronic pain is regulated by previously overlooked cells or structures in the epidermis.

There are new avenues of research on pain and movement that explore the roles of ectodermal epidermal cells.

## Data Availability

The original contributions presented in the study are included in the article/Supplementary Material, further inquiries can be directed to the corresponding author.
