# Supplementary material for: Effects on perceived pain and somatosensory function after transcutaneous neuromodulation in patients with chronic low back pain: a quasi-experimental study with a crossover intervention
Source: Front Pain Res (Lausanne). 2025 Apr 15;6:1525964. doi: 10.3389/fpain.2025.1525964 (PMC12037630; doi:10.3389/fpain.2025.1525964)
Supplement: Supplementary file 1 [file Table1.docx]

**Supplementary material. Table 1:** Pressure pain threshold at each vertebral level.

|  | **Baseline** | **Experimental tape** | **Placebo tape** | **^a^p value** | **Kendall´s W (95%CI)** |
| --- | --- | --- | --- | --- | --- |
| C3 maximum pressure (kg) to achieve PPT | 4.07 [3.07, 5.47] | 5.17 [3.95, 7.20] | 4.03 [3.10, 5.45] | X2(2)=19.858, p<0.001 | 0.255 (0.092, 0.484) |
| C3 3 kg | 1.00 [0.00, 4.00] | 0.00 [0.00, 2.50] | 1.00 [0.00, 4.50] | X2(2)=15.129, p=0.001 | 0.194 (0.019, 0.432) |
| C3 2 kg | 0.00 [0.00, 3.00] | 0.00 [0.00, 1.00] | 0.00 [0.00, 2.50] | X2(2)=11.844, p=0.003 | 0.152 (0.03, 0.375) |
| C3 1 kg | 0.00 [0.00, 0.50] | 0.00 [0.00, 0.00] | 0.00 [0.00, 1.50] | X2(2)=10.216, p=0.006 | 0.131 (0.018, 0.302) |
| C4 maximum pressure (kg) to achieve PPT | 4.10 [3.55, 5.23] | 5.50 [4.20, 6.90] | 4.20 [3.55, 5.25] | X2(2)=28.165, p<0.001 | 0.361 (0.141, 0.673) |
| C4 3 kg | 0.00 [0.00, 3.50] | 0.00 [0.00, 2.00] | 0.00 [0.00, 3.00] | X2(2)=1.755, p=0.416 | 0.022 (0.002, 0.168) |
| C4 2 kg | 0.00 [0.00, 2.00] | 0.00 [0.00, 0.50] | 0.00 [0.00, 1.00] | X2(2)=2.324, p=0.313 | 0.03 (0.001, 0.183) |
| C4 1 kg | 0.00 [0.00, 0.00] | 0.00 [0.00, 0.00] | 0.00 [0.00, 0.00] | X2(2)=0.839, p=0.657 | 0.011 (0.001, 0.134) |
| C5 maximum pressure (kg) to achieve PPT | 5.00 [4.05, 6.50] | 6.00 [4.50, 8.07] | 5.03 [4.05, 6.50] | X2(2)=16.264, p<0.001 | 0.209 (0.045, 0.503) |
| C5 3 kg | 0.00 [0.00, 3.00] | 0.00 [0.00, 0.50] | 0.00 [0.00, 3.00] | X2(2)=11.818, p=0.003 | 0.152 (0.023, 0.335) |
| C5 2 kg | 0.00 [0.00, 2.00] | 0.00 [0.00, 0.00] | 0.00 [0.00, 1.50] | X2(2)=9.234, p=0.01 | 0.118 (0.006, 0.301) |
| C5 1 kg | 0.00 [0.00, 0.50] | 0.00 [0.00, 0.00] | 0.00 [0.00, 0.00] | X2(2)=2.579, p=0.275 | 0.033 (0.001, 0.171) |
| C6 maximum pressure (kg) to achieve PPT | 5.00 [4.03, 7.45] | 5.87 [4.45, 7.88] | 5.20 [4.15, 7.50] | X2(2)=22.504, p<0.001 | 0.289 (0.098, 0.569) |
| C6 3 kg | 0.00 [0.00, 3.00] | 0.00 [0.00, 0.50] | 0.00 [0.00, 3.00] | X2(2)=6.76, p=0.034 | 0.087 (0.003, 0.253) |
| C6 2 kg | 0.00 [0.00, 1.00] | 0.00 [0.00, 0.00] | 0.00 [0.00, 1.00] | X2(2)=6.727, p=0.035 | 0.086 (0.003, 0.26) |
| C6 1 kg | 0.00 [0.00, 0.00] | 0.00 [0.00, 0.00] | 0.00 [0.00, 0.00] | X2(2)=3.185, p=0.203 | 0.041 (0.002, 0.168) |
| C7 maximum pressure (kg) to achieve PPT | 6.90 [4.45, 8.02] | 7.30 [5.93, 9.60] | 7.00 [4.60, 8.28] | X2(2)=13.338, p=0.001 | 0.171 (0.029, 0.352) |
| C7 3 kg | 0.00 [0.00, 2.50] | 0.00 [0.00, 1.00] | 0.00 [0.00, 3.00] | X2(2)=8.821, p=0.012 | 0.113 (0.018, 0.3) |
| C7 2 kg | 0.00 [0.00, 1.50] | 0.00 [0.00, 0.00] | 0.00 [0.00, 2.00] | X2(2)=11.854, p=0.003 | 0.152 (0.015, 0.36) |
| C7 1 kg | 0.00 [0.00, 0.00] | 0.00 [0.00, 0.00] | 0.00 [0.00, 0.00] | X2(2)=4.727, p=0.094 | 0.061 (0.004, 0.187) |
| D2 maximum pressure (kg) to achieve PPT | 6.10 [4.20, 9.32] | 7.20 [5.60, 9.87] | 6.10 [4.22, 9.05] | X2(2)=27.403, p<0.001 | 0.351 (0.157, 0.581) |
| D2 3 kg | 0.00 [0.00, 2.50] | 0.00 [0.00, 1.50] | 0.00 [0.00, 2.50] | X2(2)=3.2, p=0.202 | 0.041 (0, 0.297) |
| D2 2 kg | 0.00 [0.00, 1.00] | 0.00 [0.00, 0.00] | 0.00 [0.00, 1.00] | X2(2)=16, p<0.001 | 0.205 (0.103, 0.344) |
| D2 1 kg | 0.00 [0.00, 0.00] | 0.00 [0.00, 0.00] | 0.00 [0.00, 0.00] | X2(2)=13.455, p=0.001 | 0.172 (0.038, 0.311) |
| D3 maximum pressure (kg) to achieve PPT | 6.20 [4.10, 8.63] | 7.50 [5.30, 10.00] | 6.50 [4.23, 8.65] | X2(2)=26.426, p<0.001 | 0.339 (0.163, 0.578) |
| D3 3 kg | 0.00 [0.00, 4.00] | 0.00 [0.00, 1.00] | 0.00 [0.00, 3.50] | X2(2)=15.429, p<0.001 | 0.198 (0.029, 0.409) |
| D3 2 kg | 0.00 [0.00, 2.00] | 0.00 [0.00, 0.00] | 0.00 [0.00, 2.00] | X2(2)=19.37, p<0.001 | 0.248 (0.06, 0.427) |
| D3 1 kg | 0.00 [0.00, 1.00] | 0.00 [0.00, 0.00] | 0.00 [0.00, 0.50] | X2(2)=17.657, p<0.001 | 0.226 (0.102, 0.343) |
| D4 maximum pressure (kg) to achieve PPT | 6.00 [4.60, 9.20] | 6.80 [5.58, 9.73] | 6.00 [4.50, 8.90] | X2(2)=11.756, p=0.003 | 0.151 (0.013, 0.345) |
| D4 3 kg | 0.00 [0.00, 4.00] | 0.00 [0.00, 1.00] | 0.00 [0.00, 4.00] | X2(2)=18.61, p<0.001 | 0.239 (0.063, 0.424) |
| D4 2 kg | 0.00 [0.00, 3.00] | 0.00 [0.00, 0.00] | 0.00 [0.00, 2.50] | X2(2)=21.96, p<0.001 | 0.282 (0.103, 0.473) |
| D4 1 kg | 0.00 [0.00, 1.00] | 0.00 [0.00, 0.00] | 0.00 [0.00, 1.00] | X2(2)=17.333, p<0.001 | 0.222 (0.091, 0.379) |
| D5 maximum pressure (kg) to achieve PPT | 6.20 [4.85, 8.25] | 8.10 [5.50, 9.70] | 6.20 [4.80, 8.32] | X2(2)=31.312, p<0.001 | 0.401 (0.167, 0.675) |
| D5 3 kg | 0.00 [0.00, 3.00] | 0.00 [0.00, 2.00] | 0.00 [0.00, 2.50] | X2(2)=10.679, p=0.005 | 0.137 (0.021, 0.393) |
| D5 2 kg | 0.00 [0.00, 1.50] | 0.00 [0.00, 0.50] | 0.00 [0.00, 1.00] | X2(2)=7.167, p=0.028 | 0.092 (0.009, 0.238) |
| D5 1 kg | 0.00 [0.00, 0.00] | 0.00 [0.00, 0.00] | 0.00 [0.00, 0.00] | X2(2)=9.364, p=0.009 | 0.12 (0.04, 0.249) |
| D6 maximum pressure (kg) to achieve PPT | 6.40 [4.50, 8.85] | 8.40 [5.80, 10.00] | 6.70 [4.50, 8.85] | X2(2)=23.951, p<0.001 | 0.307 (0.147, 0.535) |
| D6 3 kg | 0.00 [0.00, 2.50] | 0.00 [0.00, 1.00] | 0.00 [0.00, 2.00] | X2(2)=0.436, p=0.804 | 0.006 (0, 0.121) |
| D6 2 kg | 0.00 [0.00, 1.00] | 0.00 [0.00, 0.00] | 0.00 [0.00, 1.00] | X2(2)=2.649, p=0.266 | 0.034 (0.001, 0.17) |
| D6 1 kg | 0.00 [0.00, 0.00] | 0.00 [0.00, 0.00] | 0.00 [0.00, 0.00] | X2(2)=2.48, p=0.289 | 0.032 (0, 0.141) |
| D7 maximum pressure (kg) to achieve PPT | 6.20 [4.98, 9.20] | 8.80 [5.93, 10.00] | 6.20 [5.10, 9.00] | X2(2)=38.032, p<0.001 | 0.488 (0.246, 0.671) |
| D7 3 kg | 0.00 [0.00, 2.50] | 0.00 [0.00, 0.50] | 0.00 [0.00, 2.00] | X2(2)=20.735, p<0.001 | 0.266 (0.068, 0.488) |
| D7 2 kg | 0.00 [0.00, 1.00] | 0.00 [0.00, 0.00] | 0.00 [0.00, 0.50] | X2(2)=11.73, p=0.003 | 0.15 (0.019, 0.314) |
| D7 1 kg | 0.00 [0.00, 0.00] | 0.00 [0.00, 0.00] | 0.00 [0.00, 0.00] | X2(2)=7.75, p=0.021 | 0.099 (0.005, 0.241) |
| D8 maximum pressure (kg) to achieve PPT | 7.00 [5.20, 9.20] | 8.80 [6.75, 10.00] | 7.20 [5.15, 9.15] | X2(2)=35.607, p<0.001 | 0.456 (0.231, 0.67) |
| D8 3 kg | 0.00 [0.00, 1.00] | 0.00 [0.00, 0.00] | 0.00 [0.00, 2.00] | X2(2)=7.87, p=0.02 | 0.101 (0.011, 0.307) |
| D8 2 kg | 0.00 [0.00, 0.00] | 0.00 [0.00, 0.00] | 0.00 [0.00, 0.00] | X2(2)=3.714, p=0.156 | 0.048 (0.003, 0.231) |
| D8 1 kg | 0.00 [0.00, 0.00] | 0.00 [0.00, 0.00] | 0.00 [0.00, 0.00] | X2(2)=2.947, p=0.229 | 0.038 (0.002, 0.15) |
| D9 maximum pressure (kg) to achieve PPT | 7.80 [5.58, 9.77] | 9.00 [7.40, 10.00] | 7.80 [5.43, 9.65] | X2(2)=24.248, p<0.001 | 0.311 (0.155, 0.514) |
| D9 3 kg | 0.00 [0.00, 0.00] | 0.00 [0.00, 0.00] | 0.00 [0.00, 0.00] | X2(2)=3.44, p=0.179 | 0.044 (0.002, 0.218) |
| D9 2 kg | 0.00 [0.00, 0.00] | 0.00 [0.00, 0.00] | 0.00 [0.00, 0.00] | X2(2)=4.909, p=0.086 | 0.063 (0.026, 0.145) |
| D9 1 kg | 0.00 [0.00, 0.00] | 0.00 [0.00, 0.00] | 0.00 [0.00, 0.00] | X2(2)=2.667, p=0.264 | 0.034 (0.013, 0.116) |
| D10 maximum pressure (kg) to achieve PPT | 6.97 [5.15, 9.00] | 8.73 [7.15, 10.00] | 7.00 [5.13, 9.50] | X2(2)=28.58, p<0.001 | 0.366 (0.174, 0.59) |
| D10 3 kg | 0.00 [0.00, 0.50] | 0.00 [0.00, 0.00] | 0.00 [0.00, 0.50] | X2(2)=8.4, p=0.015 | 0.108 (0.014, 0.322) |
| D10 2 kg | 0.00 [0.00, 0.00] | 0.00 [0.00, 0.00] | 0.00 [0.00, 0.00] | X2(2)=5.304, p=0.07 | 0.068 (0.004, 0.192) |
| D10 1 kg | 0.00 [0.00, 0.00] | 0.00 [0.00, 0.00] | 0.00 [0.00, 0.00] | X2(2)=4.625, p=0.099 | 0.059 (0.026, 0.155) |
| D11 maximum pressure (kg) to achieve PPT | 8.00 [5.40, 9.75] | 9.10 [6.63, 10.00] | 8.00 [5.60, 9.40] | X2(2)=25.966, p<0.001 | 0.333 (0.172, 0.515) |
| D11 3 kg | 0.00 [0.00, 2.50] | 0.00 [0.00, 0.00] | 0.00 [0.00, 2.00] | X2(2)=12.923, p=0.002 | 0.166 (0.036, 0.317) |
| D11 2 kg | 0.00 [0.00, 0.00] | 0.00 [0.00, 0.00] | 0.00 [0.00, 0.00] | X2(2)=10.138, p=0.006 | 0.13 (0.006, 0.318) |
| D11 1 kg | 0.00 [0.00, 0.00] | 0.00 [0.00, 0.00] | 0.00 [0.00, 0.00] | X2(2)=4.588, p=0.101 | 0.059 (0.015, 0.162) |
| D12 maximum pressure (kg) to achieve PPT | 7.90 [5.35, 9.00] | 9.00 [7.05, 10.00] | 7.90 [5.55, 9.08] | X2(2)=33.24, p<0.001 | 0.426 (0.201, 0.63) |
| D12 3 kg | 0.00 [0.00, 1.00] | 0.00 [0.00, 0.00] | 0.00 [0.00, 1.00] | X2(2)=4.905, p=0.086 | 0.063 (0.003, 0.243) |
| D12 2 kg | 0.00 [0.00, 0.00] | 0.00 [0.00, 0.00] | 0.00 [0.00, 0.00] | X2(2)=2.882, p=0.237 | 0.037 (0.002, 0.167) |
| D12 1 kg | 0.00 [0.00, 0.00] | 0.00 [0.00, 0.00] | 0.00 [0.00, 0.00] | X2(2)=0.636, p=0.727 | 0.008 (0.001, 0.11) |
| L1 maximum pressure (kg) to achieve PPT | 7.10 [5.50, 10.00] | 9.00 [7.10, 10.00] | 7.07 [5.50, 10.00] | X2(2)=28.214, p<0.001 | 0.362 (0.143, 0.625) |
| L1 3 kg | 0.00 [0.00, 1.00] | 0.00 [0.00, 0.00] | 0.00 [0.00, 1.00] | X2(2)=18, p<0.001 | 0.231 (0.066, 0.385) |
| L1 2 kg | 0.00 [0.00, 0.00] | 0.00 [0.00, 0.00] | 0.00 [0.00, 0.00] | X2(2)=9.923, p=0.007 | 0.127 (0.051, 0.275) |
| L1 1 kg | 0.00 [0.00, 0.00] | 0.00 [0.00, 0.00] | 0.00 [0.00, 0.00] | X2(2)=8.375, p=0.015 | 0.107 (0.026, 0.209) |
| L2 maximum pressure (kg) to achieve PPT | 7.00 [5.00, 8.75] | 8.90 [6.65, 10.00] | 7.00 [5.55, 8.80] | X2(2)=42.953, p<0.001 | 0.551 (0.387, 0.703) |
| L2 3 kg | 0.00 [0.00, 3.00] | 0.00 [0.00, 0.00] | 0.00 [0.00, 3.00] | X2(2)=21.88, p<0.001 | 0.281 (0.075, 0.433) |
| L2 2 kg | 0.00 [0.00, 1.00] | 0.00 [0.00, 0.00] | 0.00 [0.00, 1.00] | X2(2)=19.366, p<0.001 | 0.248 (0.108, 0.407) |
| L2 1 kg | 0.00 [0.00, 0.00] | 0.00 [0.00, 0.00] | 0.00 [0.00, 0.00] | X2(2)=5.846, p=0.054 | 0.075 (0.007, 0.215) |
| L3 maximum pressure (kg) to achieve PPT | 6.40 [5.23, 7.55] | 8.70 [6.70, 10.00] | 6.50 [5.40, 7.53] | X2(2)=39.511, p<0.001 | 0.507 (0.298, 0.719) |
| L3 3 kg | 2.00 [0.00, 3.00] | 0.00 [0.00, 0.50] | 2.00 [0.00, 3.50] | X2(2)=26.6, p<0.001 | 0.341 (0.144, 0.578) |
| L3 2 kg | 0.00 [0.00, 2.00] | 0.00 [0.00, 0.00] | 0.00 [0.00, 2.00] | X2(2)=25.475, p<0.001 | 0.327 (0.164, 0.519) |
| L3 1 kg | 0.00 [0.00, 0.00] | 0.00 [0.00, 0.00] | 0.00 [0.00, 0.00] | X2(2)=3.63, p=0.163 | 0.046 (0.001, 0.179) |
| L4 maximum pressure (kg) to achieve PPT | 6.50 [4.95, 7.35] | 8.00 [6.50, 9.40] | 6.70 [5.05, 7.80] | X2(2)=23.956, p<0.001 | 0.307 (0.141, 0.574) |
| L4 3 kg | 2.00 [0.00, 4.50] | 0.00 [0.00, 1.50] | 2.00 [0.00, 4.00] | X2(2)=23.863, p<0.001 | 0.306 (0.113, 0.539) |
| L4 2 kg | 1.00 [0.00, 2.00] | 0.00 [0.00, 0.00] | 0.00 [0.00, 2.00] | X2(2)=16.029, p<0.001 | 0.205 (0.03, 0.414) |
| L4 1 kg | 0.00 [0.00, 1.00] | 0.00 [0.00, 0.00] | 0.00 [0.00, 1.00] | X2(2)=7.316, p=0.026 | 0.094 (0.012, 0.271) |
| L5 maximum pressure (kg) to achieve PPT | 6.20 [4.30, 8.00] | 8.60 [7.10, 10.00] | 6.40 [4.50, 7.90] | X2(2)=47.496, p<0.001 | 0.609 (0.469, 0.811) |
| L5 3 kg | 2.00 [0.00, 5.00] | 0.00 [0.00, 2.50] | 2.00 [0.00, 5.00] | X2(2)=31.523, p<0.001 | 0.404 (0.168, 0.675) |
| L5 2 kg | 1.00 [0.00, 3.00] | 0.00 [0.00, 0.50] | 1.00 [0.00, 3.50] | X2(2)=30.185, p<0.001 | 0.387 (0.22, 0.574) |
| L5 1 kg | 0.00 [0.00, 1.00] | 0.00 [0.00, 0.00] | 0.00 [0.00, 0.50] | X2(2)=6.465, p=0.039 | 0.083 (0.007, 0.298) |
| S1 maximum pressure (kg) to achieve PPT | 7.60 [5.18, 8.85] | 9.50 [6.95, 10.00] | 7.20 [5.15, 9.00] | X2(2)=56.424, p<0.001 | 0.723 (0.612, 0.838) |
| S1 3 kg | 2.00 [0.00, 3.00] | 0.00 [0.00, 0.00] | 2.00 [0.00, 3.00] | X2(2)=41.486, p<0.001 | 0.532 (0.411, 0.674) |
| S1 2 kg | 0.00 [0.00, 1.00] | 0.00 [0.00, 0.00] | 0.00 [0.00, 1.50] | X2(2)=22.619, p<0.001 | 0.29 (0.162, 0.41) |
| S1 1 kg | 0.00 [0.00, 0.00] | 0.00 [0.00, 0.00] | 0.00 [0.00, 0.00] | X2(2)=4.727, p=0.094 | 0.061 (0.008, 0.213) |
| S2 maximum pressure (kg) to achieve PPT | 7.87 [6.05, 9.50] | 9.80 [7.42, 10.00] | 8.00 [6.05, 9.50] | X2(2)=32.118, p<0.001 | 0.412 (0.19, 0.632) |
| S2 3 kg | 0.00 [0.00, 3.00] | 0.00 [0.00, 0.50] | 0.00 [0.00, 3.00] | X2(2)=17.774, p<0.001 | 0.228 (0.073, 0.452) |
| S2 2 kg | 0.00 [0.00, 0.00] | 0.00 [0.00, 0.00] | 0.00 [0.00, 1.00] | X2(2)=17.591, p<0.001 | 0.226 (0.118, 0.364) |
| S2 1 kg | 0.00 [0.00, 0.00] | 0.00 [0.00, 0.00] | 0.00 [0.00, 0.00] | X2(2)=8, p=0.018 | 0.103 (0.039, 0.208) |
| S3 maximum pressure (kg) to achieve PPT | 8.07 [5.63, 10.00] | 10.00 [7.13, 10.00] | 8.00 [5.65, 10.00] | X2(2)=34.081, p<0.001 | 0.437 (0.258, 0.68) |
| S3 3 kg | 0.00 [0.00, 0.00] | 0.00 [0.00, 0.00] | 0.00 [0.00, 0.00] | X2(2)=6.2, p=0.045 | 0.08 (0, 0.275) |
| S3 2 kg | 0.00 [0.00, 0.00] | 0.00 [0.00, 0.00] | 0.00 [0.00, 0.00] | X2(2)=6.091, p=0.048 | 0.078 (0.011, 0.249) |
| S3 1 kg | 0.00 [0.00, 0.00] | 0.00 [0.00, 0.00] | 0.00 [0.00, 0.00] | X2(2)=4.769, p=0.092 | 0.061 (0.026, 0.136) |
| S4 maximum pressure (kg) to achieve PPT | 10.00 [7.10, 10.00] | 10.00 [8.00, 10.00] | 10.00 [7.20, 10.00] | X2(2)=2.88, p=0.237 | 0.037 (0.002, 0.167) |
| S4 3 kg | 0.00 [0.00, 0.00] | 0.00 [0.00, 0.00] | 0.00 [0.00, 0.00] | X2(2)=4, p=0.135 | 0.051 (0.026, 0.128) |
| S4 2 kg | 0.00 [0.00, 0.00] | 0.00 [0.00, 0.00] | 0.00 [0.00, 0.00] | X2(2)=4, p=0.135 | 0.051 (0.026, 0.169) |
| S4 1 kg | 0.00 [0.00, 0.00] | 0.00 [0.00, 0.00] | 0.00 [0.00, 0.00] | X2(2)=2, p=0.368 | 0.026 (0.026, 0.109) |

Data expressed with median [interquartile range]; PPT: Pressure pain threshold; 95%CI: 95% confidence interval.

^a^significant if p<0.05 (shown in red).

**Supplementary material. Table 2:** Pressure pain threshold pairwise comparisons.

|  | **Experimental - Baseline** | | **Placebo - Baseline** | | **Placebo - Experimental** | |
| --- | --- | --- | --- | --- | --- | --- |
|  | **^a^p value** | **Average difference (95%CI)** | **^a^p value** | **Average difference (95%CI)** | **^a^p value** | **Average difference (95%CI)** |
| **Averaged maximum pressure to achieve pressure pain threshold** | | | | | | |
| Cervical | <0.001 | 1 (1, 1) | >0.999 | 0.04 (-0.06, 0.15) | <0.001 | -2.507 (-3.38, -1.77) |
| Thoracic | <0.001 | 1 (1, 1) | >0.999 | 0.042 (-0.05, 0.138) | <0.001 | -3.033 (-3.821, -2.308) |
| Lumbar | <0.001 | 1 (1, 1) | 0.072 | 0.13 (0.02, 0.32) | <0.001 | -4.09 (-5.27, -3.12) |
| Sacrum | <0.001 | 1 (1, 1) | 0.197 | -0.088 (-0.212, 0.012) | <0.001 | -3.138 (-3.925, -2.3) |
| **Averaged pressure pain threshold by kilogram** | | | | | | |
| Cervical 3 kg | 0.001 | -2.4 (-4.2, -1.2) | >0.999 | 0 (-0.6, 0.6) | 0.001 | 1 (1, 1) |
| Cervical 2 kg | 0.005 | -2.1 (-3.6, -0.9) | >0.999 | -0.3 (-0.9, 0.2) | 0.005 | 1 (0.9, 1) |
| Cervical 1 kg | 0.211 | -1.7 (-3, 0) | >0.999 | 0.05 (-1.2, 0.6) | 0.173 | 1 (0, 1) |
| Thoracic 3 kg | <0.001 | -1.99 (-3, -1.25) | >0.999 | -0.083 (-0.375, 0.167) | <0.001 | 1 (1, 1) |
| Thoracic 2 kg | <0.001 | -1.53 (-2.5, -0.792) | >0.999 | 0.125 (-0.375, 0.625) | <0.001 | 1 (1, 1) |
| Thoracic 1 kg | 0.004 | -2.75 (-5.4, -1.375) | 0.146 | -1.167 (-2.575, 0) | <0.001 | 1 (0.625, 1) |
| Lumbar 3 kg | <0.001 | -3.6 (-4.8, -2.7) | >0.999 | 0.2 (-0.6, 0.8) | <0.001 | 1 (1, 1) |
| Lumbar 2 kg | <0.001 | -2.7 (-3.3, -2.1) | >0.999 | 0 (-0.6, 0.6) | <0.001 | 1 (1, 1) |
| Lumbar 1 kg | 0.004 | -2.7 (-6.95, -1.5) | 0.302 | -0.9 (-2.4, 0.6) | 0.011 | 1 (0.6, 1) |
| Sacrum 3 kg | <0.001 | -3 (-3.75, -2.25) | 0.66 | -0.375 (-1.5, 0.75) | <0.001 | 1 (1, 1) |
| Sacrum 2 kg | 0.01 | -2.25 (-3.75, -1.125) | 0.257 | 0.75 (0, 1) | 0.002 | 1 (1, 1) |
| Sacrum 1 kg | 0.1 | -2.108 (-5.25, 0) | >0.999 | -0.229 (-2.625, 1) | 0.039 | 1 (0.625, 1) |
| **Pressure pain threshold at each vertebral level** | | | | | | |
| C3 maximum pressure (kg) to achieve PPT | <0.001 | 1 (1, 1) | >0.999 | 0 (-0.3, 0.35) | <0.001 | -3.3 (-4.65, -2.15) |
| C3 3 kg | 0.038 | -4.5 (-7.5, -1.5) | >0.999 | 0 (-3, 1) | 0.044 | 1 (1, 1) |
| C3 2 kg | 0.034 | -3 (-7.5, -2) | >0.999 | -0.5 (-3, 1) | 0.049 | 1 (1, 1) |
| C3 1 kg | 0.106 | -9 (-13.75, -1.5) | 0.787 | 1 (-0.25, 1) | 0.04 | 1 (1, 1) |
| C4 maximum pressure (kg) to achieve PPT | <0.001 | 1 (1, 1) | 0.201 | 0.3 (0, 0.55) | <0.001 | -3.45 (-4.7, -2.15) |
| C4 3 kg | 0.509 | -3 (-9, 1) | >0.999 | -6 (-6, -6) | 0.843 | 1 (-3, 1) |
| C4 2 kg | 0.428 | -3 (-9, 1) | >0.999 | -6 (-6, -6) | >0.999 | 1 (-1.5, 1) |
| C4 1 kg | >0.999 | -1.5 (-7, 1) | >0.999 | -2.5 (-2.5, -2.5) | >0.999 | 0 (-4.5, 1) |
| C5 maximum pressure (kg) to achieve PPT | <0.001 | 1 (1, 1) | 0.274 | 0.2 (0, 0.45) | 0.001 | -3.25 (-4.3, -1.5) |
| C5 3 kg | 0.037 | -6 (-12, -1.5) | 0.359 | -3 (-6, 1) | 0.066 | 1 (1, 1) |
| C5 2 kg | 0.078 | -6 (-10.5, -1.5) | 0.267 | -3 (-3, -3) | 0.161 | 1 (0, 1) |
| C5 1 kg | >0.999 | -3 (-10.5, 1) | >0.999 | -3 (-6, 1) | >0.999 | 1 (-7.5, 1) |
| C6 maximum pressure (kg) to achieve PPT | 0.003 | 1 (1, 1) | >0.999 | 0.15 (-0.2, 0.55) | 0.013 | -1.95 (-3.45, -0.9) |
| C6 3 kg | 0.039 | -6 (-10.5, -1.5) | >0.999 | 0 (-3, 1) | 0.024 | 1 (1, 1) |
| C6 2 kg | 0.106 | -4.5 (-7.5, 0) | >0.999 | -1.037 (-6, 1) | 0.183 | 1 (0, 1) |
| C6 1 kg | 0.228 | -4.5 (-15, 0) | >0.999 | -3 (-3, -3) | 0.605 | 1 (-4.5, 1) |
| C7 maximum pressure (kg) to achieve PPT | 0.002 | 1 (1, 1) | >0.999 | 0.15 (-0.3, 0.75) | 0.003 | -2.85 (-4.65, -1.25) |
| C7 3 kg | 0.163 | -4.5 (-9, 0) | 0.518 | 1 (-0.5, 1) | 0.046 | 1 (1, 1) |
| C7 2 kg | 0.077 | -4.5 (-9, -1.5) | >0.999 | 0 (0, 0) | 0.079 | 1 (1, 1) |
| C7 1 kg | 0.312 | -9 (-15, 1) | >0.999 | 0.515 (1, 1) | 0.148 | 1 (1, 1) |
| D2 maximum pressure (kg) to achieve PPT | <0.001 | 1 (1, 1) | 0.291 | 0.3 (-0.05, 0.6) | <0.001 | -3.15 (-4.65, -1.85) |
| D2 3 kg | 0.179 | -4 (-6, 1) | NA |  | 0.179 | 1 (-1, 1) |
| D2 2 kg | 0.026 | -4 (-6, -2) | NA |  | 0.026 | 1 (1, 1) |
| D2 1 kg | 0.066 | -6.23 (-6, -2.333) | >0.999 | -1 | 0.064 | 1 (1, 1) |
| D3 maximum pressure (kg) to achieve PPT | <0.001 | 1 (1, 1) | >0.999 | 0.15 (-0.15, 0.5) | <0.001 | -3.6 (-5.35, -1.95) |
| D3 3 kg | 0.01 | -7.5 (-10.5, -3) | >0.999 | -1.5 (-1.5, -1.5) | 0.012 | 1 (1, 1) |
| D3 2 kg | 0.006 | -6 (-9, -3.5) | >0.999 | 1 (-3, 1) | 0.005 | 1 (1, 1) |
| D3 1 kg | 0.011 | -6 (-9, -4.5) | >0.999 | -3 (-4.5, 0) | 0.026 | 1 (1, 1) |
| D4 maximum pressure (kg) to achieve PPT | 0.002 | 1 (1, 1) | >0.999 | 0.1 (-0.3, 0.45) | 0.002 | -2.7 (-4.15, -1.2) |
| D4 3 kg | 0.009 | -7.5 (-9, -3) | >0.999 | 1 (-3, 1) | 0.005 | 1 (1, 1) |
| D4 2 kg | 0.006 | -7.5 (-9, -4.5) | >0.999 | 0.515 (1, 1) | 0.005 | 1 (1, 1) |
| D4 1 kg | 0.011 | -6 (-10.5, -4.5) | >0.999 | 0 (0, 1) | 0.01 | 1 (1, 1) |
| D5 maximum pressure (kg) to achieve PPT | <0.001 | 1 (1, 1) | >0.999 | 0.1 (-0.3, 0.65) | 0.001 | -3.15 (-4.8, -2.1) |
| D5 3 kg | 0.044 | -4.5 (-7.5, -1.5) | 0.521 | -6.728 (-3, -3) | 0.24 | 1 (0, 1) |
| D5 2 kg | 0.058 | -4.5 (-9, -1.5) | >0.999 | -2.035 (-9, 1) | 0.242 | 1 (0, 1) |
| D5 1 kg | 0.044 | -3 (-3, -3) | >0.999 | -4.5 (-4.5, -4.5) | 0.359 | 1 (-3, 1) |
| D6 maximum pressure (kg) to achieve PPT | <0.001 | 1 (1, 1) | >0.999 | 0.15 (-0.4, 0.6) | <0.001 | -3.45 (-5.2, -2.1) |
| D6 3 kg | >0.999 | -1.5 (-6, 1) | >0.999 | 0 (-3, 1) | >0.999 | 1 (-3, 1) |
| D6 2 kg | >0.999 | -1.5 (-4, 1) | >0.999 | 1 | 0.958 | 1 (-3, 1) |
| D6 1 kg | >0.999 | -1.5 (-4.5, 1) | >0.999 | 0.515 (1, 1) | >0.999 | 1 (0, 1) |
| D7 maximum pressure (kg) to achieve PPT | <0.001 | 1 (1, 1) | >0.999 | 0.15 (-0.25, 0.45) | <0.001 | -4.35 (-6.15, -2.5) |
| D7 3 kg | 0.011 | -6 (-10.5, -3) | >0.999 | -4.269 (-9, 1) | 0.013 | 1 (1, 1) |
| D7 2 kg | 0.019 | -6 (-12, -3) | >0.999 | -3 (-9, 1) | 0.035 | 1 (1, 1) |
| D7 1 kg | 0.171 | -6.75 (-8, 0) | >0.999 | -6 | 0.268 | 1 (-1, 1) |
| D8 maximum pressure (kg) to achieve PPT | <0.001 | 1 (1, 1) | 0.077 | 0.3 (0.05, 0.55) | <0.001 | -4.115 (-5.3, -2.85) |
| D8 3 kg | 0.101 | -4.5 (-10.5, 0) | 0.609 | 1 (0, 1) | 0.041 | 1 (1, 1) |
| D8 2 kg | 0.698 | -3.812 (-13.5, 1) | >0.999 | 1 (-6, 1) | 0.324 | 1 (-1.5, 1) |
| D8 1 kg | 0.503 | -3 (-9, 0) | >0.999 | -1.971 (1, 1) | 0.509 | 1 (-3, 1) |
| D9 maximum pressure (kg) to achieve PPT | <0.001 | 1 (1, 1) | >0.999 | 0 (-0.35, 0.6) | <0.001 | -3.9 (-5.8, -2.65) |
| D9 3 kg | 0.516 | -3 (-6, 0.5) | 0.521 | -2.586 (-3, -3) | 0.843 | 1 (-3, 1) |
| D9 2 kg | 0.544 | -6 (-9, -3) | >0.999 | -1.5 (-1.5, -1.5) | 0.521 | 1 (1, 1) |
| D9 1 kg | >0.999 | -4.5 (-4.5, -4.5) | >0.999 | 0 (0, 0) | >0.999 | 1 (1, 1) |
| D10 maximum pressure (kg) to achieve PPT | <0.001 | 1 (1, 1) | 0.144 | 0.25 (0, 0.55) | <0.001 | -3.7 (-5.35, -2.1) |
| D10 3 kg | 0.263 | -4.5 (-9, 1) | >0.999 | -3 (-3, -3) | 0.262 | 1 (-3, 1) |
| D10 2 kg | 0.503 | -3 (-6, 1) | >0.999 | -0.235 (-9, 1) | 0.606 | 1 (-1.5, 1) |
| D10 1 kg | 0.267 | -3 (-3, -3) | >0.999 | 0 (-3, 1) | 0.521 | 1 (1, 1) |
| D11 maximum pressure (kg) to achieve PPT | 0.002 | 1 (1, 1) | >0.999 | 0 (-0.35, 0.45) | 0.002 | -3.15 (-4.5, -1.9) |
| D11 3 kg | 0.037 | -7.5 (-12, -3) | 0.699 | -3 (-3, -3) | 0.047 | 1 (1, 1) |
| D11 2 kg | 0.145 | -4.5 (-9, 0) | >0.999 | 0 (0, 0) | 0.145 | 1 (0, 1) |
| D11 1 kg | 0.267 | -6 (-6, -6) | >0.999 | -0.515 (-3, -3) | 0.403 | 1 (1, 1) |
| D12 maximum pressure (kg) to achieve PPT | <0.001 | 1 (1, 1) | 0.074 | 0.3 (0.1, 0.55) | <0.001 | -4.2 (-5.4, -2.85) |
| D12 3 kg | 0.779 | -3 (-7.5, 1) | 0.808 | -4.5 (-12, 1) | >0.999 | 1 (-3, 1) |
| D12 2 kg | >0.999 | -3 (-6, 1) | >0.999 | -2.452 (-6, 1) | >0.999 | 1 (-3, 1) |
| D12 1 kg | >0.999 | 0 (-3, 1) | >0.999 | -3 | >0.999 | 0 (-3, 1) |
| L1 maximum pressure (kg) to achieve PPT | 0.001 | 1 (1, 1) | >0.999 | 0.1 (-0.3, 0.6) | <0.001 | -4.2 (-5.3, -3.15) |
| L1 3 kg | 0.017 | -4 (-7, -3) | NA |  | 0.017 | 1 (1, 1) |
| L1 2 kg | 0.059 | -4.5 (-9, -0.5) | >0.999 | 0.506 (-3, 1) | 0.06 | 1 (1, 1) |
| L1 1 kg | 0.17 | -6 (-6, -2) | >0.999 | -3 | 0.284 | 1 (1, 1) |
| L2 maximum pressure (kg) to achieve PPT | <0.001 | 1 (1, 1) | 0.434 | 0.3 (-0.1, 0.95) | <0.001 | -4.1 (-5.1, -3) |
| L2 3 kg | 0.005 | -6 (-9, -3) | >0.999 | -1.971 (1, 1) | 0.007 | 1 (1, 1) |
| L2 2 kg | 0.007 | -6 (-9, -3) | >0.999 | 0 (-4.5, 1) | 0.01 | 1 (1, 1) |
| L2 1 kg | 0.098 | -4.5 (-7.5, 0) | >0.999 | 0 (-3, 1) | 0.167 | 1 (1, 1) |
| L3 maximum pressure (kg) to achieve PPT | <0.001 | 1 (1, 1) | 0.042 | 0.3 (0.05, 0.6) | <0.001 | -5.7 (-7.2, -4.1) |
| L3 3 kg | 0.001 | -9 (-12, -6) | 0.609 | 1 (0, 1) | 0.001 | 1 (1, 1) |
| L3 2 kg | 0.003 | -6 (-7.5, -3) | 0.896 | 1 (0, 1) | 0.003 | 1 (1, 1) |
| L3 1 kg | 0.219 | -5.5 (-9, -1.5) | >0.999 | 0 (-6, 1) | 0.171 | 1 (0, 1) |
| L4 maximum pressure (kg) to achieve PPT | <0.001 | 1 (1, 1) | 0.205 | 0.3 (0, 0.6) | 0.002 | -4.5 (-6.75, -2.1) |
| L4 3 kg | 0.034 | -7.5 (-9, -4.5) | >0.999 | -3 (-3, -3) | 0.038 | 1 (1, 1) |
| L4 2 kg | 0.014 | -6 (-7.5, -1.5) | >0.999 | -3 (-4.5, 0) | 0.025 | 1 (1, 1) |
| L4 1 kg | 0.232 | -3 (-5, 0) | 0.447 | 1 | 0.127 | 1 (0, 1) |
| L5 maximum pressure (kg) to achieve PPT | <0.001 | 1 (1, 1) | 0.715 | 0.2 (-0.15, 0.45) | <0.001 | -6.5 (-8.15, -4.65) |
| L5 3 kg | <0.001 | -6 (-7.5, -4.5) | >0.999 | 0 (-6, 1) | 0.001 | 1 (1, 1) |
| L5 2 kg | 0.004 | -6 (-7.5, -3.5) | 0.269 | 1 (1, 1) | 0.004 | 1 (1, 1) |
| L5 1 kg | 0.521 | -3 (-6, 1) | >0.999 | -3 (-3, -3) | >0.999 | 1 (-3, 1) |
| S1 maximum pressure (kg) to achieve PPT | <0.001 | 1 (1, 1) | 0.433 | -0.3 (-0.65, 0.1) | <0.001 | -4.8 (-6, -3.75) |
| S1 3 kg | <0.001 | -7.5 (-9, -6) | >0.999 | -1.059 (-3, 1) | <0.001 | 1 (1, 1) |
| S1 2 kg | 0.007 | -6 (-9, -4.5) | >0.999 | 1 (1, 1) | 0.004 | 1 (1, 1) |
| S1 1 kg | 0.403 | -6 (-9, -1.5) | >0.999 | 0.506 (-3, 1) | 0.22 | 1 (-0.5, 1) |
| S2 maximum pressure (kg) to achieve PPT | <0.001 | 1 (1, 1) | 0.391 | 0.1 (-0.05, 0.35) | <0.001 | -3.6 (-5.25, -2.4) |
| S2 3 kg | 0.013 | -6 (-7.5, -3) | >0.999 | -2.452 (-6, 1) | 0.02 | 1 (1, 1) |
| S2 2 kg | 0.038 | -4.5 (-7.5, -3) | 0.329 | 1 (0, 1) | 0.003 | 1 (1, 1) |
| S2 1 kg | 0.17 | -6 (-9, -3) | >0.999 | -3 (-9, 1) | 0.143 | 1 (1, 1) |
| S3 maximum pressure (kg) to achieve PPT | <0.001 | 1 (1, 1) | 0.148 | -0.3 (-0.6, 0) | <0.001 | -3.9 (-5.7, -2.9) |
| S3 3 kg | 0.336 | -7.5 (-7, 1) | >0.999 | -6 | 0.509 | 1 (-2, 1) |
| S3 2 kg | >0.999 | -7.5 (-6, 1) | >0.999 | 1 | 0.703 | 1 (-3, 1) |
| S3 1 kg | 0.293 | -5.706 (-6, -3) | >0.999 | -2.452 (-6, 1) | >0.999 | 1 (1, 1) |
| S4 maximum pressure (kg) to achieve PPT | 0.116 | 1 (0.3, 1) | >0.999 | -0.05 (-2.6, 0.3) | 0.079 | -2.55 (-4.65, -0.55) |
| S4 3 kg | 0.742 | -5 (-5, -5) | NA |  | 0.742 | 1 (1, 1) |
| S4 2 kg | 0.692 | -2 | NA |  | 0.692 | 1 |
| S4 1 kg | >0.999 | -3 | >0.999 | 1 | >0.999 | 1 |

PPT: Pressure pain threshold; 95%CI: 95% confidence interval.

^a^significant if p<0.05 (shown in red).

**Supplementary material. Table 3:** Between vertebral regions pairwise comparisons p values^a^.

|  | **Baseline at 1 kg** | **Experimental tape at 1 kg** | **Placebo tape at 1 kg** | **Baseline at 2 kg** | **Experimental tape at 2 kg** | **Placebo tape at 2 kg** | **Baseline at 3 kg** | **Experimental tape at 3 kg** | **Placebo tape at 3 kg** | **Baseline at maximum pressure (kg) to achieve PPT** | **Experimental tape at maximum pressure (kg) to achieve PPT** | **Placebo tape at maximum pressure (kg) to achieve PPT** |
| --- | --- | --- | --- | --- | --- | --- | --- | --- | --- | --- | --- | --- |
| Lower thoracic vs. Cervical | <0.001 | <0.001 | <0.001 | <0.001 | 0.001 | <0.001 | <0.001 | <0.001 | <0.001 | <0.001 | <0.001 | <0.001 |
| Lumbar vs. Cervical | >0.999 | >0.999 | >0.999 | 0.02 | >0.999 | >0.999 | >0.999 | <0.001 | >0.999 | >0.999 | >0.999 | 0.015 |
| Lumbar vs. Lower thoracic | <0.001 | <0.001 | <0.001 | 0.655 | 0.008 | 0.038 | <0.001 | >0.999 | <0.001 | <0.001 | <0.001 | 0.791 |
| Sacrum vs. Cervical | 0.225 | 0.037 | 0.907 | <0.001 | 0.651 | 0.062 | 0.174 | <0.001 | 0.185 | 0.192 | 0.61 | <0.001 |
| Sacrum vs. Lower thoracic | >0.999 | 0.151 | 0.004 | >0.999 | >0.999 | >0.999 | >0.999 | >0.999 | 0.413 | 0.003 | 0.002 | >0.999 |
| Sacrum vs. Lumbar | 0.225 | 0.001 | 0.022 | 0.158 | >0.999 | >0.999 | 0.055 | 0.846 | 0.09 | 0.004 | 0.024 | 0.415 |
| Upper thoracic vs. Cervical | 0.202 | 0.004 | 0.216 | 0.016 | 0.023 | 0.01 | 0.098 | 0.001 | 0.017 | 0.003 | 0.147 | 0.015 |
| Upper thoracic vs. Lower thoracic | 0.053 | 0.005 | 0.006 | 0.009 | >0.999 | 0.115 | 0.067 | 0.007 | 0.049 | 0.003 | 0.001 | 0.006 |
| Upper thoracic vs. Lumbar | 0.202 | 0.001 | 0.003 | >0.999 | 0.13 | >0.999 | 0.014 | >0.999 | 0.003 | <0.001 | 0.006 | >0.999 |
| Upper thoracic vs. Sacrum | >0.999 | >0.999 | >0.999 | 0.087 | >0.999 | >0.999 | >0.999 | 0.108 | >0.999 | >0.999 | >0.999 | 0.111 |

^a^significant if p<0.05 (shown in red).

**Supplementary material. Table 4:** Within legs pairwise comparisons in Lunge test.

|  | **Right leg** | | **Left leg** | |
| --- | --- | --- | --- | --- |
|  | **^a^p value** | **Average difference (95%CI)** | **^a^p value** | **Average difference (95%CI)** |
| Experimental tape - Baseline | <0.001 | 1 (1, 1) | <0.001 | 1 (1, 1) |
| Placebo tape - Baseline | 0.027 | 1 (0.5, 1) | 0.976 | 0.75 (-0.75, 1) |
| Placebo tape - Experimental tape | <0.001 | -4.5 (-6, -3.75) | <0.001 | -5.25 (-6.75, -4.5) |

95%CI: 95% confidence interval.

^a^significant if p<0.05 (shown in red).
